# Trade-off between synergy and efficacy in combinations of HIV-1 latency-reversing agents

**DOI:** 10.1371/journal.pcbi.1006004

**Published:** 2018-02-16

**Authors:** Vipul Gupta, Narendra M. Dixit

**Affiliations:** 1 Department of Chemical Engineering, Indian Institute of Science, Bangalore, India; 2 Centre for Biosystems Science and Engineering, Indian Institute of Science, Bangalore, India; Icahn School of Medicine at Mount Sinai, UNITED STATES

## Abstract

Eradicating HIV-1 infection is difficult because of the reservoir of latently infected cells that gets established soon after infection, remains hidden from antiretroviral drugs and host immune responses, and retains the capacity to reignite infection following the cessation of treatment. Drugs called latency-reversing agents (LRAs) are being developed to reactivate latently infected cells and render them susceptible to viral cytopathicity or immune killing. Whereas individual LRAs have failed to induce adequate reactivation, pairs of LRAs have been identified recently that act synergistically and hugely increase reactivation levels compared to individual LRAs. The maximum synergy achievable with LRA pairs is of clinical importance, as it would allow latency-reversal with minimal drug exposure. Here, we employed stochastic simulations of HIV-1 transcription and translation in latently infected cells to estimate this maximum synergy. We incorporated the predominant mechanisms of action of the two most promising classes of LRAs, namely, protein kinase C agonists and histone deacetylase inhibitors, and quantified the activity of individual LRAs in the two classes by mapping our simulations to corresponding *in vitro* experiments. Without any adjustable parameters, our simulations then quantitatively captured experimental observations of latency-reversal when the LRAs were used in pairs. Performing simulations representing a wide range of drug concentrations, we estimated the maximum synergy achievable with these LRA pairs. Importantly, we found with all the LRA pairs we considered that concentrations yielding the maximum synergy did not yield the maximum latency-reversal. Increasing concentrations to increase latency-reversal compromised synergy, unravelling a trade-off between synergy and efficacy in LRA combinations. The maximum synergy realizable with LRA pairs would thus be restricted by the desired level of latency-reversal, a constrained optimum we elucidated with our simulations. We expect this trade-off to be important in defining optimal LRA combinations that would maximize synergy while ensuring adequate latency-reversal.

## Author summary

HIV-1 infection typically requires lifelong treatment because a class of infected cells called latently infected cells remains hidden from drugs and host immune responses and can reignite infection when treatment is stopped. Massive efforts are ongoing to devise ways to eliminate latently infected cells. The most advanced of the strategies developed for this purpose involves using drugs called latency-reversing agents (LRAs), which reactivate latently infected cells, effectively bringing them out of their hiding. Multiple mechanisms are involved in the establishment of latency. Pairs of LRAs targeting distinct mechanisms have been found to synergize and induce significantly higher latency-reversal than individual LRAs. If this synergy can be maximized, latency-reversal can be achieved with minimal drug exposure. Using stochastic simulations of HIV-1 latency, we unraveled a trade-off between synergy and efficacy in LRA pairs. Drug concentrations that maximized synergy did not also maximize latency-reversal. Drug concentrations that yielded higher latency-reversal compromised synergy and vice versa. This trade-off would constrain the synergy realizable between LRAs and guide the identification of optimal LRA combinations that would maximize synergy while ensuring adequate latency-reversal.

## Introduction

Combination antiretroviral therapy (cART) for HIV-1 infection can suppress the viral load in infected individuals to below the detection limit but is unable to eradicate the virus [1]. The key obstacle to achieving sterilizing cure is the presence of a reservoir of long-lived latently infected cells that cannot be eliminated by cART [2] and can reignite infection upon the cessation of therapy [3]. Latently infected cells harbor replication-competent integrated HIV-1 genomes that remain transcriptionally silent, escaping the action of antiretroviral drugs and immune recognition [4]. The reservoir is thought to be established soon after infection [5] and is estimated to have a half-life of many years [2, 6]. Cells in the reservoir are thought to be activated stochastically [7] and can reignite infection often within weeks [8] but sometimes years after the cessation of therapy [9], necessitating lifelong therapy. Significant efforts are ongoing, therefore, to quantify the size of the reservoir, define the type and location of cells constituting it, and devise ways of eliminating it [10, 11].

The most advanced of the strategies to eliminate the latent reservoir, called the “shock and kill” strategy, advocates the use of drugs called latency-reversing agents (LRAs) which stimulate HIV-1 transcription in latently infected cells, rendering them susceptible to viral cytopathicity or immune recognition and killing [11–13]. Several classes of LRAs have been developed, each targeting one or more of the mechanisms underlying viral latency [14]. Multiple cellular and viral mechanisms have been implicated in the establishment of latency, including cytoplasmic localization of the transcription factor NF-κB, sequestration of the protein complex P-TEFb involved in transcription, and epigenetic silencing due to acetylation and methylation [10]. Additional mechanisms involving possible hardwiring of latency in the HIV-1 genome [15] as well as the prevention of latency-reversal by the mTOR complex [16] have recently been identified. LRAs called PKC agonists stimulate the PKC pathway leading among other things to the upregulation and enhanced nuclear translocation of NF-κB [17]. Another class of LRAs called histone deacetylase inhibitors (HDACi’s) induces chromatin remodeling, accelerating HIV-1 transcriptional elongation [18]. Other classes of LRAs include histone methyltransferase inhibitors (HMTi’s), DNA methyltransferase inhibitors (DNMTi’s), and bromodomain and extraterminal (BET) domain inhibitors, which induce HIV-1 transcription via other proposed mechanisms [14].

Several LRAs have been tested extensively *in vitro* and *ex vivo* and some have reached clinical trials [19–24]. LRAs have induced transient viral production in infected individuals but have failed to lower the size of the latent reservoir [12]. Individual LRAs have been shown *ex vivo* to be grossly inadequate at reversing latency when compared to the maximal reversal achieved with agents inducing global T cell activation [25, 26]. Furthermore, a single pulse of even the highest activating dose of the latter agents seems to reactivate only a subset of the latently infected cells *in vitro* [4]. Serial stimulation does seem to increase the fraction of cells reactivated [27], indicating that multiple doses of an LRA may be necessary to achieve the desired latency-reversal *in vivo*. Recent studies have therefore examined latency-reversal with combinations of LRAs acting via distinct mechanisms and identified, promisingly, combinations that can reactivate cells *ex vivo* to extents comparable to that achieved with global T cell activation [28, 29]. With the use of multiple drugs, however, toxicities may become limiting. LRAs that act synergistically, reactivating more cells together than expected based on their individual potencies, are therefore of particular interest as they may achieve the desired latency-reversal with the least drug exposure. Indeed, several *in vitro* and *ex vivo* studies have identified LRA combinations that act synergistically [28–39]. An important goal that follows is to determine the concentrations of the LRAs in these promising combinations that would lead to the maximum synergy.

In the present study, we employed stochastic simulations of HIV-1 transcription and translation in infected cells, *i*.*e*., the so-called intracellular HIV-1 latency circuit, to quantify the activities of individual LRAs and estimate the maximum synergy realizable between pairs of LRAs. Stochastic simulations of the HIV-1 latency circuit have been employed extensively in previous studies and have provided key insights into the mechanisms underlying the establishment of latency and its reversal [7, 15, 40–43]. Importantly, they recapitulate the two distinct fates, *viz*., productive infection (or activation) and latency, realized by cells following their infection by HIV-1 [7, 15, 40–42]. Furthermore, they have elucidated the role of the viral protein Tat in inducing a stochastic switch from latency to activation [7, 43], identified a potential cause of the slow viral load decline during cART [40], and suggested the intriguing possibility of latency-reversal by enhancing noise in viral transcription [41]. Previous studies, however, have not explicitly considered the role of LRAs. An alternative class of models that considers populations of latently infected cells, often using sophisticated techniques from the theory of branching processes, has been constructed to estimate quantities of clinical interest such as the reduction in the latent reservoir that would allow a desired duration of cART interruption without viral rebound [28, 44–48]. These latter models do not consider intracellular events explicitly, precluding a description of drug synergy. Here, we built on the previous stochastic simulations of the HIV-1 latency circuit by explicitly incorporating steps affected by LRAs. We considered LRAs belonging to the two most promising classes, *viz*., HDACi’s, which have entered clinical trials [19, 20, 23], and PKC agonists, which have shown synergy with almost every other class of LRAs *in vitro* and *ex vivo* [22, 28, 31]. We quantified the activity of individual LRAs by altering the rates of the relevant steps in the HIV-1 latency circuit to match the extent of latency-reversal observed *in vitro*. Our simulations then captured quantitatively, without any adjustable parameters, the synergy observed when the LRAs were employed together, giving us confidence in our formalism. We then applied our simulations to predict the maximum synergy realizable with the combinations and the corresponding drug concentrations. We found, interestingly, that a trade-off exists between the synergy and the efficacy of LRA combinations that could introduce new limits on their usage. The maximum synergy realizable would have to be constrained by this trade-off in achieving the desired extent of latency-reversal, a consideration that may be important in defining the optimal usage of LRA combinations.

## Results

### Model of the HIV-1 latency circuit

**Fig 1:**
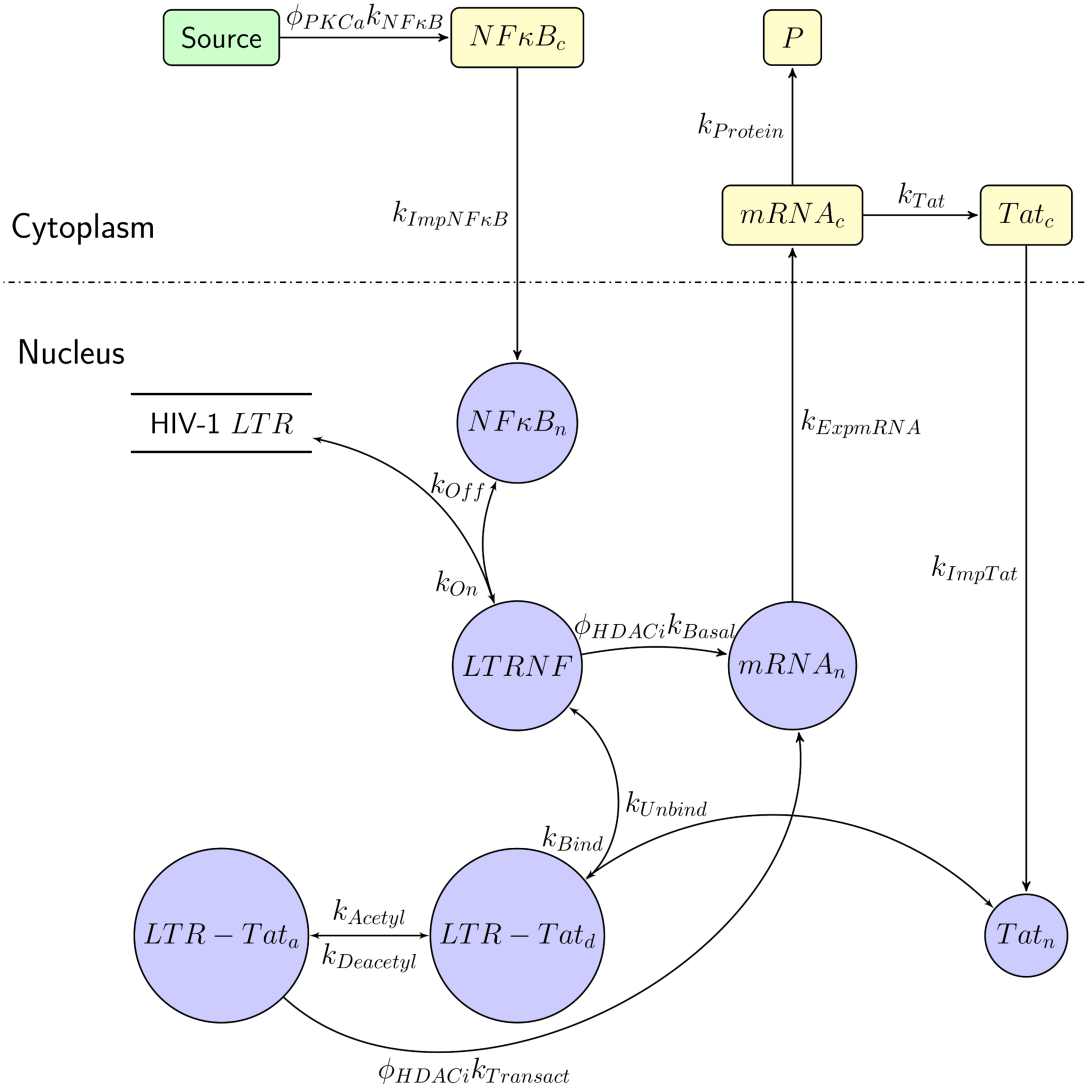
Schematic of the HIV-1 latency circuit. The events associated with HIV-1 transcription and translation that govern the latency of an HIV-1 infected cell are depicted as a set of reactions (see Eqs (1)–(18) in Methods). The entities involved in the reactions are described in Results and the rate constants of the reactions are in Table 1.

We considered the following set of events involved in HIV-1 transcription and translation in an HIV-1-infected cell harboring an integrated, replication-competent HIV-1 genome (Fig 1). The host transcription factor NF-κB is produced in the cytoplasm and is translocated to the nucleus [49]. In the nucleus, it reversibly binds the long terminal repeat (LTR) region of the integrated HIV-1 genome to form a complex [50], denoted LTRNF. This complex formation triggers HIV-1 transcription at a low, basal rate [7]. The viral mRNA thus produced is translocated to the cytoplasm, where it is translated. Translation yields the viral protein Tat as well as other viral proteins, P. Tat is translocated back to the nucleus, where it upregulates HIV-1 transcription via multiple mechanisms [51]. We focused here on its role in enhancing HIV-1 transcription by binding to the HIV-1 LTR. The binding of both NF-κB and Tat to HIV-1 LTR has been argued to be essential for enhanced HIV-1 transcription [52]. We therefore let Tat bind LTRNF to form a complex, denoted LTR-Tat_*d*_. Several other factors, including PTEF-b, are essential for transcriptional elongation [53], which we did not consider explicitly for simplicity (see Discussion) and based on previous studies where PTEF-b and the other factors are argued to be in stoichiometric excess [7, 40]. (Previous models have denoted LTR-Tat_*d*_ as PTEF-b_*d*_ [40].) Tat is typically in its deacetylated form, which in this complex can get reversibly acetylated [7], and is then denoted LTR-Tat_*a*_. The latter complex triggers HIV-1 transcription at an enhanced rate [7], following which it dissociates into its constituents. The resulting viral mRNA can produce more Tat, which in turn can further accelerate HIV-1 transcription. This positive feedback, which is triggered following a stochastic build-up of Tat beyond a threshold, drives a cell out of latency [7, 43].

LRAs facilitate this reactivation. We assumed that PKC agonists increase the production rate of NF-κB, *k*_*NFκB*_, by a factor *ϕ*_*PKCa*_ in a dose-dependent manner. HDACi’s increase the HIV-1 transcription rate in the absence and presence of Tat, denoted *k*_*Basal*_ and *k*_*Transact*_, respectively, by a factor *ϕ*_*HDACi*_, again in a dose-dependent manner. Proteins and mRNA are subject to degradation in the nucleus and the cytoplasm.

We performed stochastic simulations of the above events, constituting the HIV-1 latency circuit, using the Gillespie algorithm [54] (Methods). The parameter values employed are in Table 1.

**Table 1:** Model parameters and their typical values.

Rate constant	Event	Value	Source
*k*_*NFκB*_	Production of NF-κB	9×10^−5^ molecules s^-1^	Fit
*k*_*ImpNFκB*_	Import of NF-κB into the nucleus	9×10^−2^ s^-1^	[68]
*k*_*On*_	Association of NF-κB with HIV-1 LTR	2.1×10^−5^ molecules^-1^ s^-1^	[50]
*k*_*Off*_	Dissociation of NF-κB–LTR complex	9.9×10^−3^ s^-1^	[50]
*k*_*Basal*_	Basal transcription of HIV-1	6.1×10^−3^ s^-1^	Fit
*k*_*ExpmRNA*_	Export of HIV-1 mRNA to cytoplasm	7.2×10^−4^ s^-1^	[40]
*k*_*Protein*_	Translation of HIV-1 proteins	10^−2^ s^-1^	
*k*_*Tat*_	Translation of Tat	1.3×10^−3^ s^-1^	
*k*_*ImpTat*_	Import of Tat into the nucleus	5.1×10^−3^ s^-1^	
*k*_*Bind*_	Association of Tat with NF-κB bound LTR	1.5×10^−4^ molecules^-1^ s^-1^	
*k*_*Unbind*_	Dissociation of Tat–LTR complex	1.7×10^−2^ s^-1^	
*k*_*Acetyl*_	Acetylation of LTR associated Tat	10^−3^ s^-1^	
*k*_*Deacetyl*_	Deacetylation of LTR associated Tat	0.13 s^-1^	
*k*_*Transact*_	Tat-induced transcription of HIV-1	0.1 s^-1^	
*δ*_*mRNA*_	Degradation of HIV-1 mRNA	4.8×10^−5^ s^-1^	
*δ*_*Tat*_	Degradation of Tat	4.3×10^−5^ s^-1^	
*δ*_*Protein*_	Degradation of viral proteins	5×10^−5^ s^-1^	
*δ*_*NFκB*_	Degradation of NF-κB	2.8×10^−5^ s^-1^	[69]
*ϕ*_*PKCa*_	Fold increase in *k*_*NFκB*_ due to a PKC agonist	≥1	
*ϕ*_*HDACi*_	Fold increase in *k*_*Basal*_ and *k*_*Transact*_ due to an HDACi	≥1	

### Basal reactivation of latently infected cells

**Fig 2:**
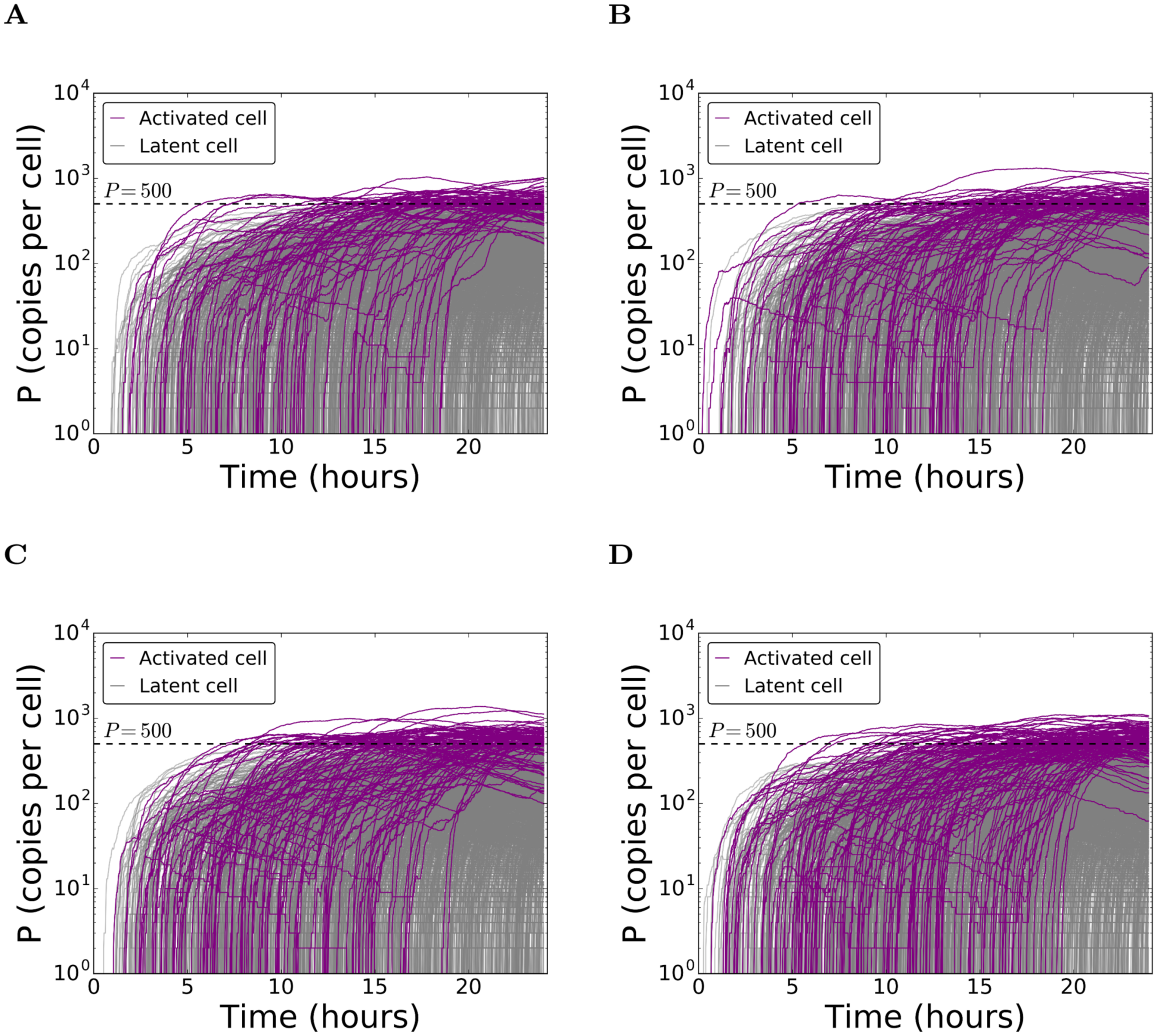
Basal reactivation of latently infected cells. Time-evolution of protein copy numbers in latently infected cells obtained by stochastic simulations of the HIV-1 latency circuit (Methods) in the absence of intervention. Trajectories (lines) of each of the 2000 cells in one realization are shown. Those crossing the activation threshold of 500 copies (dashed line) are in purple and the rest in grey. The parameters employed for the simulations except *k*_*NFκB*_ and *k*_*Basal*_ are in Table 1. The values of the latter parameters and the resulting percentage activation, *f*_*on*_, are: (A) *k*_*NFκB*_ = 9 × 10^−5^ molecules s^-1^ and *k*_*Basal*_ = 6.14 × 10^−3^ s^-1^ yielding *f*_*on*_ = 0.0395; (B) *k*_*NFκB*_ = 10^−4^ molecules s^-1^ and *k*_*Basal*_ = 6.14 × 10^−3^ s^-1^ yielding *f*_*on*_ = 0.0475; (C) *k*_*NFκB*_ = 9 × 10^−5^ molecules s^-1^ and *k*_*Basal*_ = 7 × 10^−3^ s^-1^ yielding *f*_*on*_ = 0.053; and (D) *k*_*NFκB*_ = 10^−4^ molecules s^-1^ and *k*_*Basal*_ = 7 × 10^−3^ s^-1^ yielding *f*_*on*_ = 0.0656.

We first considered the spontaneous reactivation of latently infected cells in the absence of drugs. A cell was assumed to be activated if the level of viral proteins in it rose above a threshold. To define this threshold, we performed simulations in the absence of NF-κB and Tat, where no activation of cells is expected (Methods). The highest protein levels achieved in these simulations were ~130 copies/cell (S1 Fig). We therefore set the threshold at 500 copies/cell, which could only be achieved via NF-κB- and/or Tat-mediated increase in HIV-1 transcription, indicating activation. We now performed simulations of the complete HIV-1 latency circuit (Fig 1; Eqs (1)–(18) in Methods). We set *ϕ*_*PKCa*_ = 1 and *ϕ*_*HDACi*_ = 1 to mark the absence of drugs. The simulations captured the two distinct fates achieved by infected cells, latency and activation (Fig 2A). Most of the cells remained latently infected, in consonance with experiments [29, 33]. We performed simulations with different values of *k*_*NFκB*_ and *k*_*Basal*_, which remain poorly estimated, to identify conditions that mimicked experimental observations of the fraction of cells activated, *f*_*on*_, in the absence of drugs. In the experimental data we considered (Methods), *f*_*on*_ = 0.039 ± 0.008 in 24 h following the start of the experiment [33]. We found that with *k*_*NFκB*_ = 9 × 10^−5^ molecules s^-1^ and *k*_*Basal*_ = 6.14 × 10^−3^ s^-1^, our simulations yielded *f*_*on*_ = 0.039 ± 0.003 (Fig 2A), in close agreement with the data. We employed these values for further simulations. Increasing *k*_*NFκB*_, *k*_*Basal*_ or both increased *f*_*on*_ (Fig 2B–2D). We recognized that the combination of parameter values we employed was not unique. Alternative combinations, however, did not alter our key findings (S2 Fig).

### Dose-response curves of individual drugs

**Fig 3:**
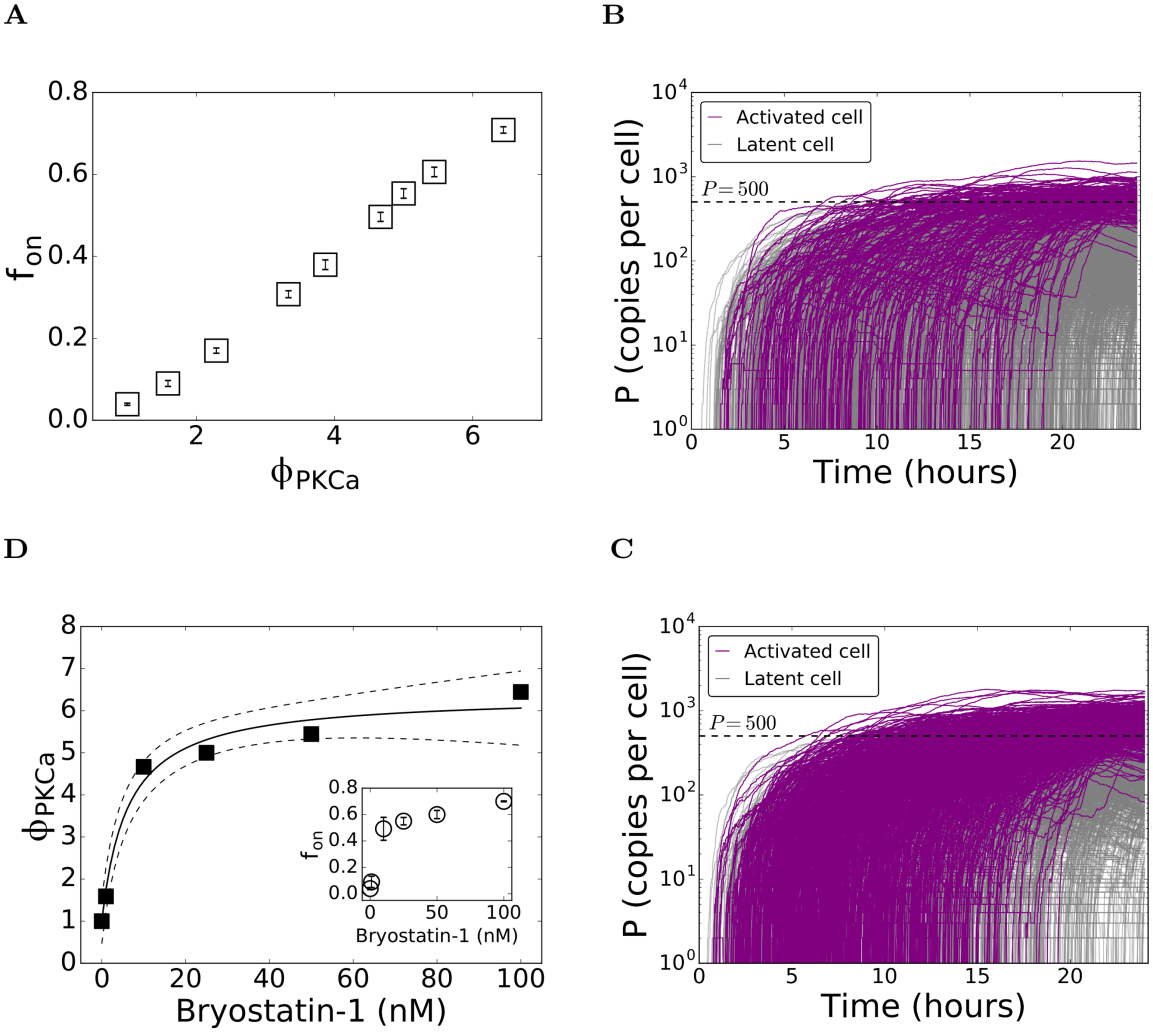
Influence of PKC agonists on latent cell reactivation. (A) The fraction of cells reactivated, *f*_*on*_, as a function of the fold-increase, *ϕ*_*PKCa*_, in the rate of NF-κB synthesis, predicted using our stochastic simulations (Methods). Representative realizations showing the time-evolution of protein copy numbers in activated (purple) and latent (grey) cells with (B) *ϕ*_*PKCa*_ = 1.76 yielding *f*_*on*_ = 0.1 and (C) *ϕ*_*PKCa*_ = 3.87 yielding *f*_*on*_ = 0.38. The remaining parameters are in Table 1. (D) Dose-response curve for bryostatin-1 obtained by mapping *ϕ*_*PKCa*_ to the dosage [D] (symbols) that yield the measured *f*_*on*_ [33] (*Inset*). The best-fit of the Hill equation (Eq (22)) (solid line) and the 95% confidence interval (dashed lines) are also shown. The best-fit parameter estimates are *ϕ*_0_ = 5.3 ± 0.3 and *ϕ*_*M*_ = 6 ± 2 nM (R^2^ = 0.98).

We next considered the influence of individual drugs by increasing either *ϕ*_*PKCa*_ or *ϕ*_*HDACi*_ while keeping all the other parameters fixed. To mimic the influence of PKC agonists, we performed simulations with different values of *ϕ*_*PKCa*_ > 1 while keeping *ϕ*_*HDACi*_ = 1. We found that *f*_*on*_ increased monotonically with *ϕ*_*PKCa*_ (Fig 3A). For instance, *f*_*on*_ = 0.1 with *ϕ*_*PKCa*_ = 1.76 and *f*_*on*_ = 0.38 with *ϕ*_*PKCa*_ = 3.87 (Fig 3B and 3C). To map our simulations to experiments, we considered data of *f*_*on*_ as a function of the concentration, [*D*], of the PKC agonist bryostatin-1 [33]. Corresponding to each value of [*D*] employed, we obtained a value of *ϕ*_*PKCa*_ from our simulations that yielded *f*_*on*_ in agreement with the experiments, thus linking our simulations to the experiments (Fig 3D). We fit a Hill equation (Eq (22)) to this data linking [*D*] to *ϕ*_*PKCa*_ and obtained a dose-response curve for bryostatin-1, which allowed estimation of *f*_*on*_ also at values of [*D*] not employed in the experiments. The equation fit the data well (Fig 3D). We found that *ϕ*_*PKCa*_ rose from 1 when [*D*] = 0 to ~4.5 when [*D*] = 10 nM. A further increase in [*D*] yielded only a marginal increase in *ϕ*_*PKCa*_; *ϕ*_*PKCa*_ appeared to saturate at ~6 (Fig 3D).

**Fig 4:**
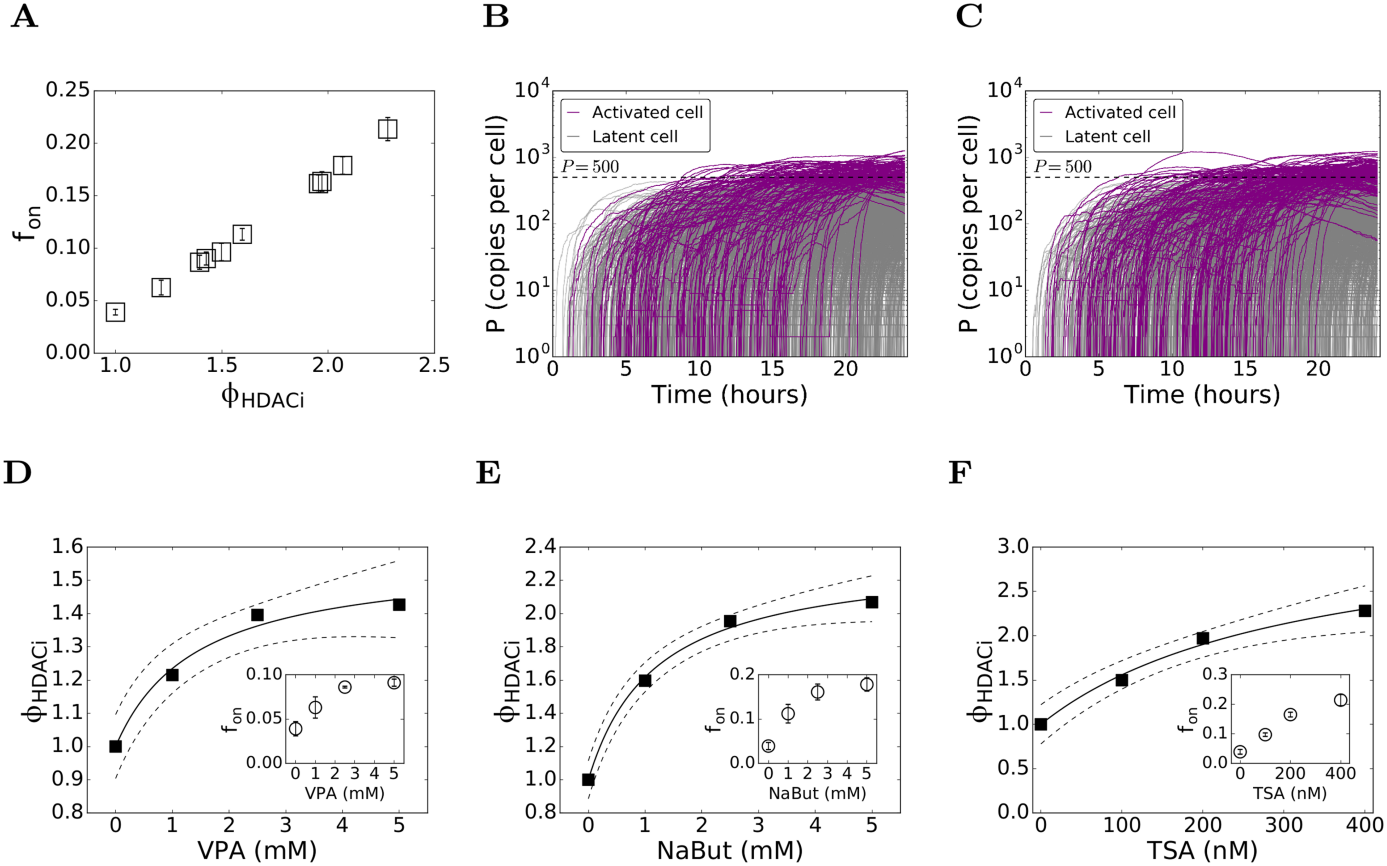
Influence of HDACi’s on latent cell reactivation. (A) The fraction of cells reactivated, *f*_*on*_, as a function of the fold-increase, *ϕ*_*HDACi*_, in the rate of HIV-1 transcription, predicted using our stochastic simulations (Methods). Representative realizations showing the time-evolution of protein copy numbers in activated (purple) and latent (grey) cells with (B) *ϕ*_*HDACi*_ = 1.24 yielding *f*_*on*_ = 0.066 and (C) *ϕ*_*HDACi*_ = 1.36 yielding *f*_*on*_ = 0.0825. The remaining parameters are in Table 1. Dose-response curves for (D) VPA, (E) NaBut, and (F) TSA, obtained by mapping *ϕ*_*HDACi*_ to the dosage [D] (symbols) that yield the measured *f*_*on*_ [33] (*Insets*). The best-fits of the Hill equation (Eq (22)) (solid line) and the 95% confidence interval (dashed lines) are also shown. The best-fit parameter estimates are (D) *ϕ*_0_ = 0.57 ± 0.08, *ϕ*_*M*_ = 1.4 ± 0.5 mM (R^2^ = 0.98); (E) *ϕ*_0_ = 1.35 ± 0.08, *ϕ*_*M*_ = 1.1 ± 0.2 mM (R^2^ = 0.99); and (F) *ϕ*_0_ = 2.3 ± 0.4, *ϕ*_*M*_ = 300 ± 100 nM (R^2^ = 0.99).

To mimic HDACi’s, we performed simulations with *ϕ*_*HDACi*_ > 1 while keeping *ϕ*_*PKCa*_ = 1. Again, *f*_*on*_ increased monotonically with *ϕ*_*HDACi*_ (Fig 4A). For instance, with *ϕ*_*HDACi*_ = 1.24, we found that *f*_*on*_ = 0.066 and with *ϕ*_*HDACi*_ = 1.36, *f*_*on*_ = 0.082 (Fig 4B and 4C). We now considered experimental data for the HDACi’s VPA, TSA, and NaBut [33]. Following the procedure above, we obtained dose-response curves for each of these drugs linking their concentrations [*D*] to *ϕ*_*HDACi*_ (Fig 4D–4F). The Hill equation again provided good fits to data of all the three drugs. We found interestingly that *ϕ*_*HDACi*_ saturated to different values for the three drugs. It saturated at ~1.5 for VPA, ~2.2 for NaBut, and ~3 for TSA. (Note that for TSA, *ϕ*_*HDACi*_ did not saturate in the concentration range studied, but the trend towards saturation was evident. The best-fit parameters of the Hill equation provided an estimate of the saturating value of *ϕ*_*HDACi*_) Ignoring toxicities, these fits thus allowed rank-ordering of the HDACi’s in terms of the maximum activation levels they can achieve individually. Accordingly, TSA>NaBut>VPA.

### Combinations of PKC agonists and HDACi’s

**Fig 5:**
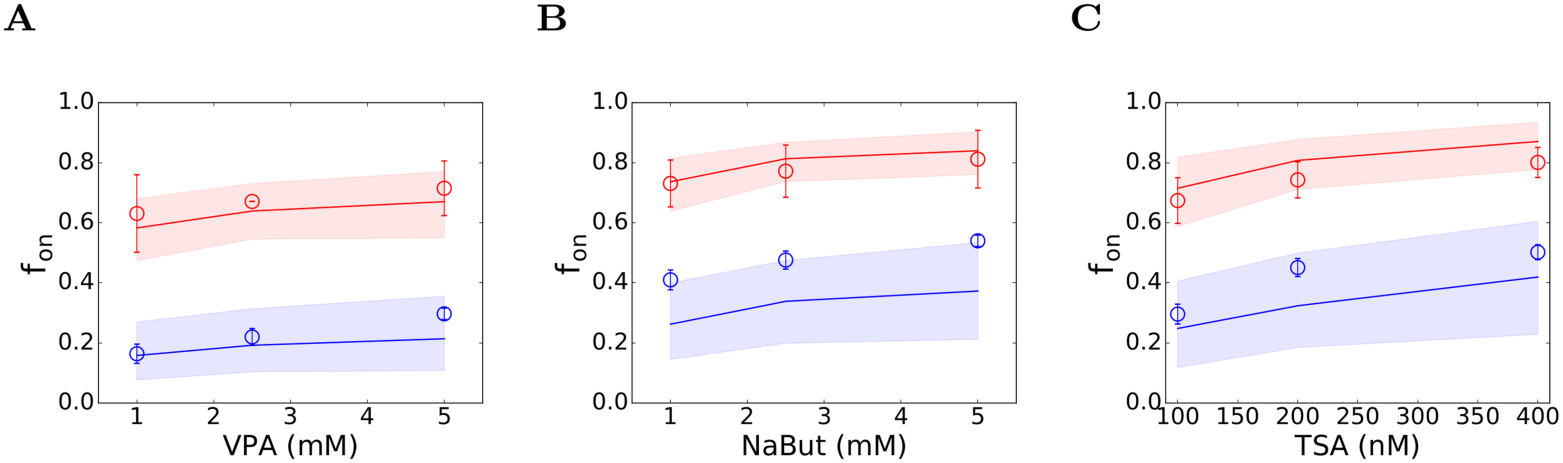
Co-stimulation with PKC agonists and HDACi’s. The fraction of cells reactivated, *f*_*on*_, following simultaneous exposure to 1 nM (blue) or 10 nM (red) bryostatin-1 and (A) VPA, (B) NaBut, and (C) TSA, observed experimentally [33] (symbols) and predicted by our simulations (lines). The values of *ϕ*_*PKCa*_ and *ϕ*_*HDACi*_ corresponding to the individual drug concentrations employed were obtained from the dose-response curves in Figs 3 and 4, respectively. Simulations based on confidence limits on these parameter values yielded 95% confidence limits on our predictions (shaded regions). All the other parameters are in Table 1.

We next predicted the influence of using bryostatin-1 together with each of the HDACi’s above and compared our predictions with experiments. Two concentrations of bryostatin-1, viz., 1 and 10 nM, and three concentrations of the HDACi’s viz., 1, 2.5, and 5 mM for VPA and NaBut and 100, 200, and 400 nM for TSA, were employed in combination in the experiments [33]. Our dose-response curves above identified the values of *ϕ*_*PKCa*_ and *ϕ*_*HDACi*_ corresponding to each of these concentrations. We employed these latter values in our simulations and predicted *f*_*on*_. We found that our predictions were in good agreement with experiments (Fig 5). With 10 nM bryostatin-1 and 2.5 mM VPA, we predicted *f*_*on*_ = 0.64, whereas the corresponding experimental observation was *f*_*on*_ = 0.67 (Fig 5A). With 10 nM bryostatin-1, our simulations were similarly in excellent agreement with data at other concentrations of VPA (Fig 5A) and all concentrations of NaBut and TSA (Fig 5B and 5C). With 1 nM bryostatin-1, our simulations were again in good agreement with data in combination with VPA and TSA, but tended to under-predict data with NaBut, where the data lay at the edge of the 95% confidence interval of our predictions. Overall, this agreement, without any adjustable parameters, was remarkable and gave us confidence in our model and our predictions. We applied it next to estimate the maximum synergy achievable between bryostatin-1 and each of these HDACi’s.

### Synergy between PKC agonists and HDACi’s

**Fig 6:**
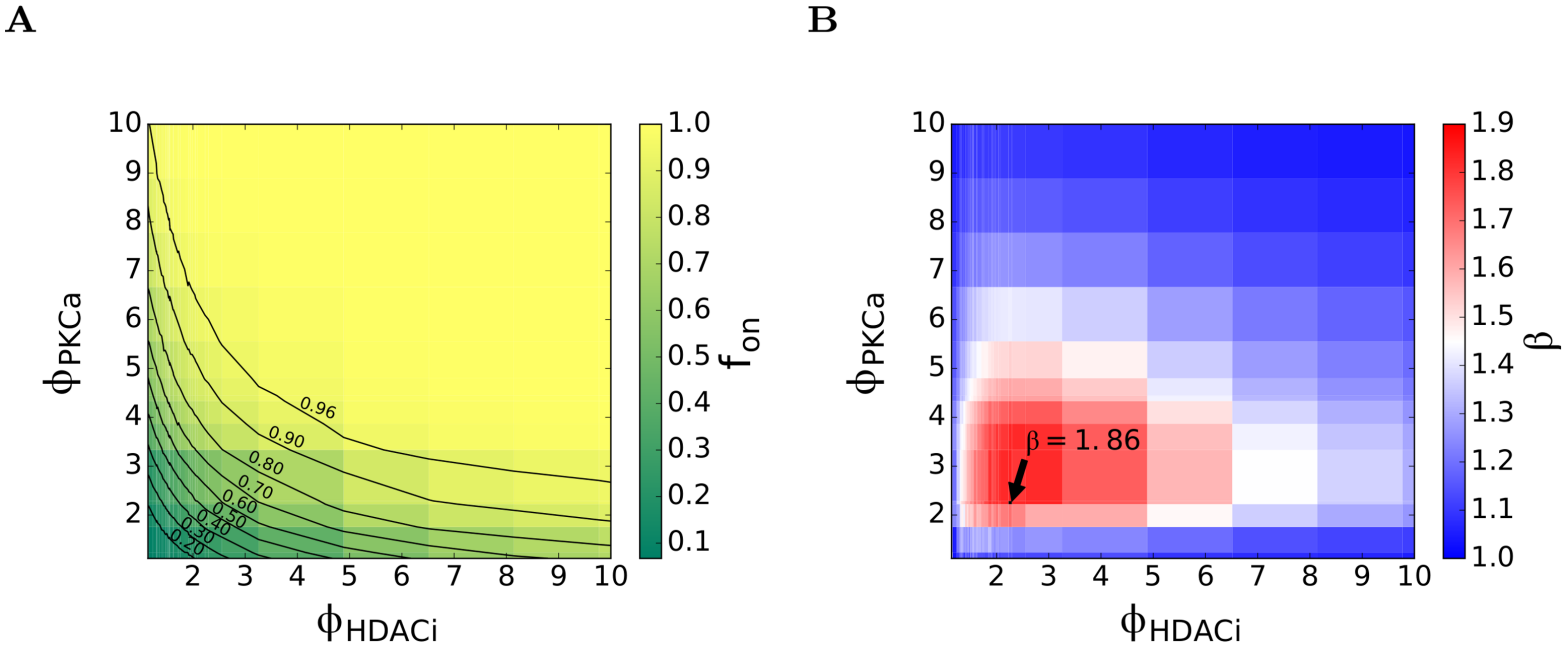
Synergy between PKC agonists and HDACi’s. (A) The fraction of cells reactivated, *f*_*on*_ predicted by our simulations for different values of *ϕ*_*PKCa*_ and *ϕ*_*HDACi*_, the fold-increase in the rate of NF-κB synthesis and HIV-1 transcription due to a PKC agonist and an HDACi, respectively. The lines are contours of constant *f*_*on*_. (B) The corresponding synergy between the drugs, *β*, predicted using Eqs (19)–(21). The maximum synergy is indicated.

We performed simulations with a range of values of *ϕ*_*PKCa*_ and *ϕ*_*HDACi*_ and estimated *f*_*on*_ and the extent of synergy, *β* (see Eq (21)), for each pair of values of *ϕ*_*PKCa*_ and *ϕ*_*HDACi*_. To encompass the range in the dose-response curves above, we let *ϕ*_*PKCa*_ and *ϕ*_*HDACi*_ both go from 1 to 10. We found that *f*_*on*_ rose from its basal value as either *ϕ*_*PKCa*_ or *ϕ*_*HDACi*_ increased and eventually reached a value of 1 at sufficiently high values of these parameters (Fig 6A). For instance, when both *ϕ*_*PKCa*_ and *ϕ*_*HDACi*_ were >5, *f*_*on*_ was nearly 1, indicating 100% activation. (Note that such high values of *ϕ*_*HDACi*_ are not realizable using the drugs we considered even at the highest doses, precluding such high activation rates.) Further, because increasing either *ϕ*_*PKCa*_ or *ϕ*_*HDACi*_ can increase *f*_*on*_, a locus of points on a *ϕ*_*PKCa*_ versus *ϕ*_*HDACi*_ plot could be identified that corresponded to a desired *f*_*on*_. For instance, the combination *ϕ*_*PKCa*_ ≈ 8 and *ϕ*_*HDACi*_ = 1 yielded *f*_*on*_ = 0.9 as did the combination *ϕ*_*PKCa*_ ≈ 2 and *ϕ*_*HDACi*_ = 10 and a number of other combinations in between, yielding the locus for *f*_*on*_ = 0.9 (Fig 6A). We thus identified loci for several values of *f*_*on*_ starting from 0.2 to 0.96 (Fig 6A).

Using the simulations above, we calculated *β* for each pair of values of *ϕ*_*PKCa*_ and *ϕ*_*HDACi*_ following Eqs (19)–(21). To calculate *β*, the increase in *f*_*on*_ over the basal level relative to the increase possible with maximal stimulation (defined by *f*_*ax*_ in Eq (19)) must be determined. In experiments, maximal stimulation was achieved by exposing cells to agents such as PMA/I, which yielded *f*_*on*_ = 0.853 [33]. The mechanisms that prevent 100% activation remain unclear. In our simulations, increasing *ϕ*_*PKCa*_ or *ϕ*_*HDACi*_ sufficiently yielded *f*_*on*_ = 1. Indeed, under such circumstances, our simulations agreed with a deterministic model of the HIV-1 latency circuit, which predicts 100% activation (S1 Text, S3 Fig). Effects, including stochastic ones, that limit activation following PMA/I exposure may thus exist upstream of the HIV-1 latency circuit we considered (for instance, in the PKC pathway leading to NF-κB synthesis). Here, based on our simulations, we let *f*_*on*_ corresponding to the maximal stimulation, termed *f*_*on*_ (*positive control*) in Eq (19), be 1.

We found that *β* ranged from 1 to ~1.9 as we spanned the range of values of *ϕ*_*PKCa*_ and *ϕ*_*HDACi*_ from 1–10 (Fig 6B). Note that *β*>1 implies synergy, *β*<1 antagonism and *β* = 1 Bliss independence. We found that *β* ≈ 1 when either *ϕ*_*PKCa*_ or *ϕ*_*HDACi*_ was very low (~1) or very high (~10). We understood these results as follows. When both *ϕ*_*PKCa*_ and *ϕ*_*HDACi*_ were low, the effect of either drug was negligible. The chance that the drugs acted on the same cell, given the strong stochastic effects, was small, precluding any synergy. When at least one of *ϕ*_*PKCa*_ and *ϕ*_*HDACi*_ was high, the corresponding drug(s) achieved nearly maximal activation, leaving little room for the other drug to exert any influence. Synergy therefore was again not possible.

The drugs interacted synergistically when *ϕ*_*PKCa*_ and *ϕ*_*HDACi*_ were both increased moderately above 1. Here, the PKC agonist increased NF-κB synthesis and its LTR binding, leaving the integrated HIV-1 genome poised for transcription. The HDACi could then accelerate transcription, triggering the Tat-mediated positive feedback and inducing activation. Indeed, we found that *β* was maximum, at ~1.9, when *ϕ*_*PKCa*_ and *ϕ*_*HDACi*_ were both ~2.2 (Fig 6B). *β* decreased as *ϕ*_*PKCa*_ and *ϕ*_*HDACi*_ either increased or decreased from these optimal values, which we visualized using a heat map (Fig 6B).

### Drug concentrations yielding maximum synergy

**Fig 7:**
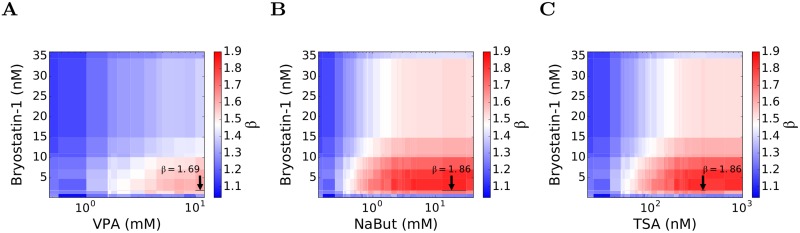
Drug concentrations yielding maximum synergy. Synergy as a function of the concentrations of bryostatin-1 and (A) VPA, (B) NaBut, and (C) TSA, obtained by mapping *ϕ*_*PKCa*_ and *ϕ*_*HDACi*_ in Fig 6B to drug concentrations using the dose-response curves in Figs 3 and 4. The maximum synergy attainable is indicated.

We next considered the specific combinations of bryostatin-1 with VPA, TSA, and NaBut. Using the dose-response curves above, we identified concentrations of bryostatin-1 corresponding to the *ϕ*_*PKCa*_ values in Fig 6B. Similarly, we identified concentrations of each of the HDACi's corresponding to the *ϕ*_*HDACi*_ values therein. We thus obtained the extent of synergy achieved at any given concentration of bryostatin-1 used in conjunction with any given concentration of any of the HDACi's. We visualized this dependence of synergy on the concentrations again using heat maps (Fig 7). We could now identify the concentrations that yielded the maximum synergy. We found that 1.83 nM of bryostatin-1 together with 12.5 mM of NaBut yielded the maximum synergy of *β*~1.9 (Fig 7B). Similarly, the same concentration of bryostatin-1 along with 353 nM of TSA yielded *β*~1.9 (Fig 7C). With VPA, however, the saturation in the dose-response curve occurred at *ϕ*_*HDACi*_ ~1.5, which was much lower than the value of ~2.2 corresponding to the maximum synergy. Thus, VPA could only achieve sub-maximal synergy compared to the other HDACi's. We found that the maximum synergy achieved between VPA and bryostatin-1 saturated at *β*~1.7, which occurred at concentrations of >5 mM and ~1.8 nM, respectively, of the two drugs (Fig 7A).

### Synergy-efficacy trade-off

**Fig 8:**
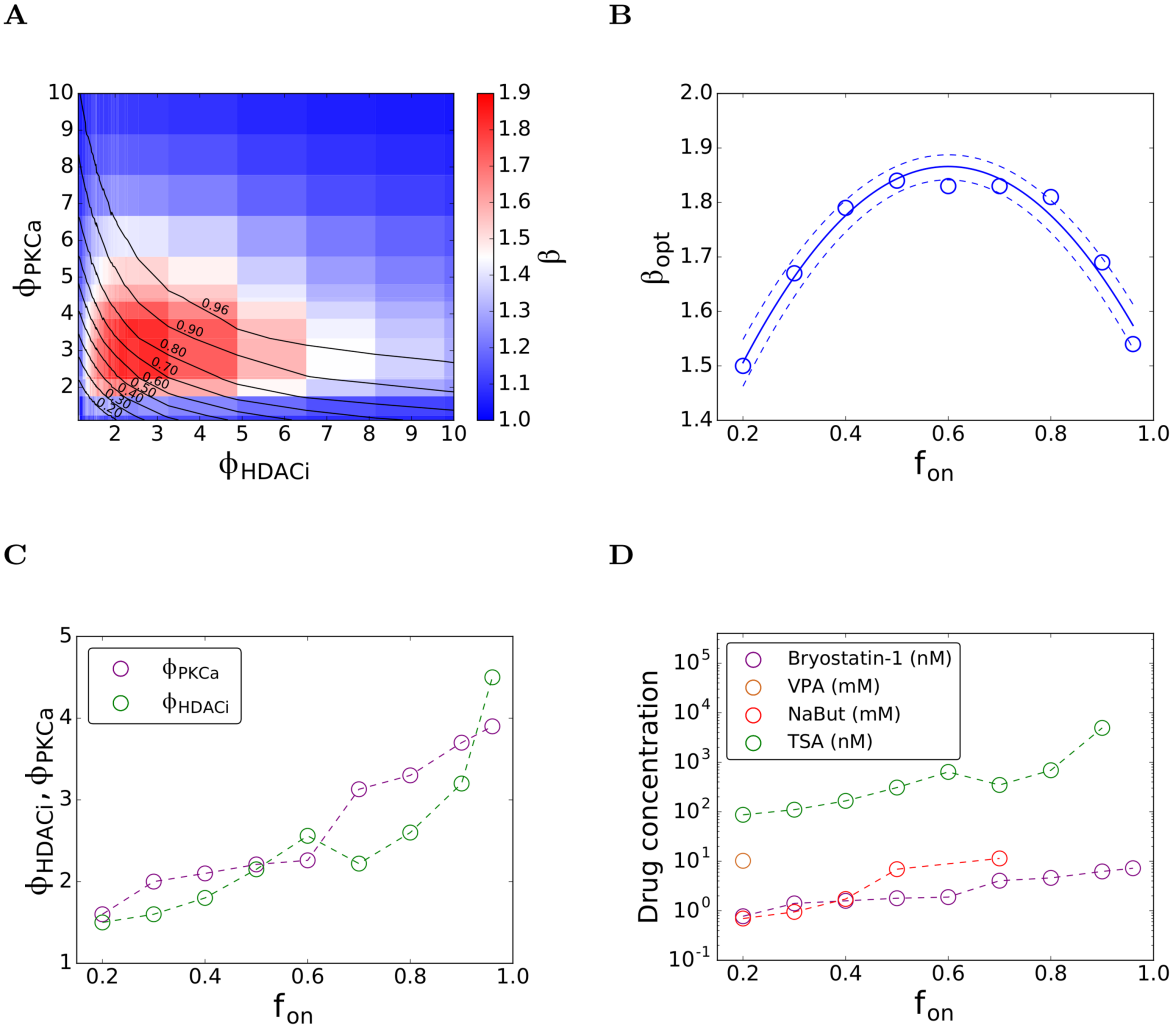
The synergy-efficacy trade-off. (A) The contours of constant *f*_*on*_ (Fig 6A) superimposed on the synergy heatmap (Fig 6B). (B) The maximum synergy as a function of *f*_*on*_ demonstrating the synergy-efficacy trade-off (symbols). The line is a quadratic fit to guide the eye. (C) The values of *ϕ*_*PKCa*_ and *ϕ*_*HDACi*_ that maximize *β* as functions of *f*_*on*_ and (D) the associated drug concentrations estimated using the dose-response curves (Figs 3 and 4).

Maximum synergy implies that the "increase" in *f*_*on*_ due to the interaction between drugs over that in the absence of any interaction is the maximum. However, the resulting *f*_*on*_ despite the maximal increase may not be adequately high. Indeed, we found that the values of *ϕ*_*PKCa*_ and *ϕ*_*HDACi*_ that yielded the maximum synergy, *β*~1.9, corresponded to *f*_*on*_~0.5 (Fig 6B). Higher drug concentrations would thus be necessary to achieve higher *f*_*on*_. The extent of synergy, however, would then be compromised. We visualized this trade-off between *β* and *f*_*on*_ by superimposing the loci of constant *f*_*on*_ on the heat map of *β* (Fig 8A). As *f*_*on*_ increased beyond ~0.5, *β* kept decreasing below 1.9.

Given this trade-off, it is of interest to determine the maximum synergy achievable while ensuring a desired *f*_*on*_. On any locus of fixed *f*_*on*_, we found that *β* was low when *ϕ*_*PKCa*_ was high and *ϕ*_*HDACi*_ was low and vice versa, whereas *β* was high at intermediate values of *ϕ*_*PKCa*_ and *ϕ*_*HDACi*_. We denoted the maximum *β* on a locus as *β*_*opt*_, to signify the optimum achieved constrained by *f*_*on*_. With *f*_*on*_ = 0.9, for instance, we found that *β*_*opt*_ was 1.69, which occurred at *ϕ*_*PKCa*_~3.7 and *ϕ*_*HDACi*_ ~3.2. We thus computed *β*_*opt*_ as a function of *f*_*on*_ (Fig 8B) and also obtained the corresponding values of *ϕ*_*PKCa*_ and *ϕ*_*HDACi*_ (Fig 8C). When *f*_*on*_ was low, *β*_*opt*_ increased with *f*_*on*_. It reached a maximum at intermediate values of *f*_*on*_ and subsequently declined for higher values of *f*_*on*_ (Fig 8B). With *f*_*on*_ = 0.96, for instance, *β*_*opt*_ was ~1.54. As expected, *ϕ*_*PKCa*_ and *ϕ*_*HDACi*_ corresponding to *β*_*opt*_ rose with *f*_*on*_ (Fig 8C). Using the dose-response curves for the individual drugs (Figs 3 and 4), we could now estimate the drug concentrations corresponding to *β*_*opt*_ as a function of *f*_*on*_ (Fig 8D). Note that drug concentrations could be estimated for values of *ϕ*_*PKCa*_ and *ϕ*_*HDACi*_ below the saturation limits in the dose-response curves; where drug effects saturate, the synergy realizable would be lower than *β*_*opt*_. We thus found that to achieve *f*_*on*_ = 0.8, for instance, we could use bryostatin-1 at 4.6 nM and TSA at 688 nM, yielding *β*_*opt*_~1.8. Any other combination of concentrations of these drugs that would yield the same *f*_*on*_ would have a lower synergy, resulting in greater drug exposure than necessary.

## Discussion

Eliminating latency in HIV-1 infection has not been possible so far except possibly with the Berlin patient, who continues to be in remission many years after a successful bone marrow transplantation [55]. Yet, that the influence of the latent reservoir can be limited to a point where adaptive immune responses can prevent full-blown infection has been demonstrated by the post-treatment control achieved by a subset of patients in the VISCONTI trial, who following early cART initiation have maintained undetectable viral load long after cessation of therapy [56, 57]. Lowering the size of the latent cell reservoir thus presents promise of durable control of infection and disease progression if not sterilizing cure. Achieving this lowering of the size of the latent reservoir too has proven a challenge *in vivo* [11, 12]. Combinations of LRAs targeting distinct mechanisms underlying HIV-1 latency have shown synergy *in vitro* and *ex vivo* in reactivating latently infected cells [28–39], presenting a potential avenue for achieving the desired reduction in the latent reservoir *in vivo*. In the present study, using stochastic simulations that quantitatively captured published *in vitro* experiments, we unraveled a trade-off between the synergy and the efficacy of LRA combinations that may constrain their *in vivo* potency and may have to be accounted for in defining optimal LRA combinations.

Our simulations build on the framework for recapitulating HIV-1 latency *in silico* employed extensively in previous studies [7, 15, 40–42]. The advance our study makes is in incorporating into the framework the specific steps in the HIV-1 latency circuit affected by two prominent classes of LRAs and describing experimental observations of latency-reversal with the LRAs and the associated synergy quantitatively. Specifically, we incorporated NF-κB synthesis, its nuclear translocation, and its binding to the HIV-1 LTR for initiating HIV-1 transcription, so that the effects of PKC agonists like bryostatin-1, which upregulate the PKC pathway and increase NF-κB synthesis [17], could be described. HDACi’s, the other class of drugs we considered, were assumed to affect the rate of HIV-1 transcription [18]. We estimated the influence of individual LRAs on these processes by matching our simulations with corresponding *in vitro* experiments. Remarkably, without any adjustable parameters, our simulations then predicted quantitatively experimental observations of the extent of latent cell reactivation when the LRAs were used in pairs, giving us confidence in our simulations. We used these simulations to predict the level of synergy expected at a host of concentrations of the drugs not employed in the experiments and identified the maximum synergy achievable.

The trade-off between synergy and efficacy we unraveled implies that at the drug concentrations that yield the maximum synergy, the extent of latent cell reactivation may not be maximal. Increasing drug concentrations could improve reactivation levels, but would compromise synergy. Maximizing synergy would minimize drug levels and hence side effects and costs. The absolute extent of reactivation, however, is likely to be more important clinically, say, for achieving post-treatment control [57]. The synergy realizable would thus have to be constrained by the extent of reactivation desired. We showed that this constrained optimum can be realized for a specified extent of reactivation by the appropriate choice of drug concentrations. Future experiments, employing a range of concentrations of LRAs used in pairs, as has been done recently to elucidate the synergy between the noise enhancer V11 and the PKC agonist prostratin [41], could serve to validate our prediction of this synergy-efficacy trade-off.

Based on our simulations, the drugs synergized because the PKC agonists upregulated NF-κB synthesis, increasing NF-κB bound to LTR, which rendered the HIV-1 genome amenable to transcription, and the HDACi’s increased the rate of this transcription. The drugs together thus led to a much higher level of HIV-1 transcription and latent cell reactivation than achieved by the drugs independently. We recognize that additional mechanisms of synergy between PKC agonists and HDACi’s have been proposed. For instance, PKC agonists may increase the production of P-TEFb and HDACi’s may facilitate the release of P-TEFb from the repressive 7SK snRNP complex [58]. Synergy has also been proposed to arise when HDACi’s increase the stochastic noise in HIV-1 gene expression and PKC agonists increase the mean HIV-1 gene expression level [41]. Tat may also contribute to the observed synergy by helping recruit the PTEF-b complex and other transcriptional elongational factors to the LTR [53, 59] or by increasing the nuclear uptake of NF-κB by freeing it from the inhibitory molecule IκB [60]. We did not consider these latter phenomena explicitly in our simulations. Our goal was to construct a minimal model of the HIV-1 latency circuit that could describe the influence of LRA combinations. That our simulations captured experimental observations quantitatively, without any adjustable parameters, indicated that our formalism was robust; the influence of the phenomena we ignored was either small in the experiments we considered or was suitably subsumed in our simulations.

We recognize that toxicity can be a key limitation in defining optimal LRA combinations. Previous studies have suggested strategies to optimize treatments based on the trade-off between efficacy and toxicity (e.g., [61]). Our study presents an additional factor, the synergy-efficacy trade-off, that is expected to be important in optimizing LRA treatments. Where toxicity is not limiting, the synergy-efficacy trade-off may be the defining factor in optimizing treatments. For instance, in vitro studies report that bryostatin-1 is not toxic up to concentrations of 10 nM [33], well above the concentrations (~2 nM) at which our simulations predict the maximum synergy. Where toxicity is significant, a more comprehensive formalism that integrates the synergy-efficacy trade-off with the toxicity-efficacy trade-off would serve to identify optimal LRA combinations. Constructing such a formalism presents a promising avenue for future studies.

Although *in vitro* studies, on which our simulations were based, have been widely used to test drug effects and have led to the identification of LRAs and their synergistic combinations (e.g., [29, 41, 61]; reviewed in [62]), they do not recapitulate all the complexities of the scenario *in vivo* [25]. For instance, the extent of reactivation of latent cells appears much higher *in vitro* than *ex vivo*, the latter more akin to the scenario *in vivo* [25, 26]. Our findings thus remain to be established *ex vivo*. Using our simulations to mimic *ex vivo* data was precluded by the large variations in the data introduced possibly by inter-patient differences; even with exposure to global T cell activating stimuli, which should induce maximal latent cell reactivation, a variation of >2 logs in the fold-induction in intracellular HIV-1 RNA and >3 logs in supernatant HIV-1 RNA levels has been observed *ex vivo* [28, 29]. The origins of these large variations remain to be fully understood. Nonetheless, we applied our simulations to *in vitro* data of 3 pairs of LRAs and found the trade-off between synergy and efficacy to be relevant to all the pairs, suggesting that our findings are likely to be more widely applicable, extending to *ex vivo* and *in vivo* settings and to other classes of LRAs. We therefore expect the synergy-efficacy trade-off to become a potentially important factor in defining optimal LRA combinations.

## Methods

### Stochastic simulations of the HIV-1 latency circuit

The events constituting the HIV-1 latency circuit (Fig 1) are listed schematically as reactions along with their rate constants in the equations below (Eqs (1)–(18)). Events representing the degradation of the entities involved are also included.

$$\Phi \overset{\phi_{PKCa}k_{NF\kappa B}}{\to }NF-\kappa B_{c}$$ (1)

$$NF-\kappa B_{c}\overset{k_{ImpNF\kappa B}}{\to }NF-\kappa B_{n}$$ (2)

$$LTR+NF-\kappa B_{n}\overset{k_{On}}{\underset{k_{Off}}{⇄}}LTRNF$$ (3)

$$LTRNF\overset{\phi_{HDACi}k_{Basal}}{\to }LTRNF+mRNA_{n}$$ (4)

$$mRNA_{n}\overset{k_{ExpmRNA}}{\to }mRNA_{c}$$ (5)

$$mRNA_{c}\overset{k_{Protein}}{\to }mRNA_{c}+P$$ (6)

$$mRNA_{c}\overset{k_{Tat}}{\to }mRNA_{c}+Tat_{c}$$ (7)

$$Tat_{c}\overset{k_{ImpTat}}{\to }Tat_{n}$$ (8)

$$LTRNF+Tat_{n}\overset{k_{Bind}}{\underset{k_{Unbind}}{⇄}}LTR-Tat_{d}$$ (9)

$$LTR-Tat_{d}\overset{k_{Acetyl}}{\underset{k_{Deacetyl}}{⇄}}LTR-Tat_{a}$$ (10)

$$LTR-Tat_{a}\overset{\phi_{HDACi}k_{Transact}}{\to }LTR+mRNA_{n}+NF-\kappa B_{n}+Tat_{n}$$ (11)

$$NF-\kappa B_{c}\overset{\delta_{NF\kappa B}}{\to }\Phi$$ (12)

$$NF-\kappa B_{n}\overset{\delta_{NF\kappa B}}{\to }\Phi$$ (13)

$$mRNA_{c}\overset{\delta_{mRNA}}{\to }\Phi$$ (14)

$$mRNA_{n}\overset{\delta_{mRNA}}{\to }\Phi$$ (15)

$$P\overset{\delta_{Protein}}{\to }\Phi$$ (16)

$$Tat_{c}\overset{\delta_{Tat}}{\to }\Phi$$ (17)

$$Tat_{n}\overset{\delta_{Tat}}{\to }\Phi$$ (18)

We performed stochastic simulations of the above system of events using the Gillespie algorithm implemented in the tool StochKit [63]. The rate constants were obtained from the literature (Table 1). The production rate of NF-κB, *k*_*NFκB*_, and the basal transcription rate of HIV-1, *k*_*Basal*_, remained uncertain. We fixed them to mimic experiments (see Results). We set the initial copy numbers of all species to zero, except LTR, which we set to 1, recognizing that a vast majority of infected cells harbors single proviruses [64]. Mimicking experiments [33], we ran the simulations for 24 h. If the protein level, P, in a cell crossed a threshold (see below), we considered the cell to have been activated. We performed simulations with 2000 cells and computed the fraction of cells that thus got activated. We repeated the simulations 10 times and obtained the average fraction of cells activated, which we denoted *f*_*on*_. Increasing the number of cells or realizations did not affect our results (S4 Fig).

To define the activation threshold, we performed the above simulations in the absence of NF-κB and Tat. The positive feedback leading to enhanced HIV-1 transcription then does not occur and cells are bound to remain latent. We therefore set the activation threshold to be sufficiently higher than the maximum protein level achieved in these simulations. We ensured that variation in this threshold did not affect our results (S2 Fig).

### The influence of LRAs and synergy

We performed simulations with *ϕ*_*PKCa*_ = 1 and *ϕ*_*HDACi*_ = 1 to mimic experiments without drugs and with *ϕ*_*PKCa*_ > 1 and *ϕ*_*HDACi*_ >1 to mimic the scenario in the presence of PKC agonists and HDACi’s, respectively. To quantify the influence of drugs, we followed previous formalisms [28, 33, 61] and defined *f*_*on*_(*no drug*) and *f*_*on*_ (*positive control*) as the activation level without drugs and with maximal stimulation. The normalized fraction of cells activated by drug ‘x’ was then given by

$$f_{ax}=\frac{f_{on}(drug\;x)-f_{on}(no\;drug)}{f_{on}(positive\;control)-f_{on}(no\;drug)}$$ (19)

The expected activation level if the two drugs were to act independently was “predicted” by the Bliss independence model:

$$f_{axy,P}=f_{ax}+f_{ay}-f_{ax}f_{ay}$$ (20)

Here, *f*_*ax*_ was obtained by setting *ϕ*_*PKCa*_ > 1 and *f*_*ay*_ by setting *ϕ*_*HDACi*_ > 1, respectively. To mimic the use of the drugs simultaneously, we set both *ϕ*_*PKCa*_ > 1 and *ϕ*_*HDACi*_ > 1 and obtained the “observed” activation level, *f*_*axy*,*O*_. To quantify the interactions between the drugs, we computed

$$\beta =\frac{f_{axy,O}}{f_{axy,P}}$$ (21)

If *β >* 1, the drugs act synergistically, whereas if *β* < 1, they act antagonistically. *β* = 1 would imply Bliss independence, *i*.*e*., the absence of any interaction between the drugs.

### Comparisons with data

We considered the previously reported *in vitro* data on the activation of latently infected cells exposed to the PKC agonist bryostatin-1 and the HDACi’s VPA, NaBut, and TSA [33]. To compare our simulations with the data, we first considered data with single drugs. For each drug concentration employed, we identified a value of *ϕ* (either *ϕ*_*PKCa*_ or *ϕ*_*HDACi*_) that yielded *f*_*on*_ in agreement with the corresponding experimental observation. For bryostatin-1, we thus obtained values of *ϕ*_*PKCa*_ for the various drug levels employed. Similarly, for each of the HDACi’s we obtained values of *ϕ*_*HDACi*_ corresponding to the various drug levels employed. We then used each pair of these *ϕ*_*PKCa*_ and *ϕ*_*HDACi*_ values in our simulations to predict *f*_*on*_ when the drugs were used together.

### Optimum synergy

We performed simulations with different combinations of *ϕ*_*PKCa*_ and *ϕ*_*HDACi*_, each spanning the range from 1 to 10, and estimated *β* (Eq (21)). The combination of values yielding the maximum *β* yielded the maximum synergy. Simultaneously, the values of *f*_*on*_ allowed elucidation of the synergy-efficacy trade-off. To estimate drug concentrations corresponding to any specified *β* and *f*_*on*_, we constructed dose-response curves for each drug as follows. We employed the estimates of *ϕ* we obtained above corresponding to the concentrations used in the experiments and fit the modified Hill equation,

$$\phi =1+\frac{\phi_{0}[D]}{\phi_{M}+[D]}$$ (22)

to the data, where [*D*] was the drug concentration, using *ϕ*_0_ and *ϕ*_*M*_ as adjustable parameters. Note that the modified Hill equation above is identical in form to the standard E-max model of the influence of drugs [65]. The resulting dose-response curves then allowed us to identify drug concentrations that would yield the desired *β* and *f*_*on*_, including the maximum synergy. We identified these concentrations for bryostatin-1 and each of the three HDACi’s.

We performed data analysis using Python 2.7 [66] and plotted using Matplotlib 1.5.1 [67].

## Supporting information

S1 Text Deterministic model of the HIV-1 latency circuit. (PDF)

S1 FigThreshold protein copy number for activation.Protein copy numbers in cells lacking Tat and NF-κB obtained by simulating the reduced latency circuit, $mRNA_{c}\overset{k_{Protein}}{\to }P+mRNA_{c}$, $mRNA_{c}\overset{\delta_{mRNA}}{\to }\Phi$ and $P\overset{\delta_{Protein}}{\to }\Phi$, which captures the protein production following a single stochastic transcription event yielding a copy of HIV-1 mRNA. The simulations were thus performed with the initial conditions *mRNA*(0) = 1 and *P*(0) = 0. The mean (line) and standard deviation (shaded region) of the resulting time-evolution of *P* from 10^4^ realizations (or cells) is shown, establishing a lower bound on the threshold *P* for reactivation of latently infected cells.(TIF)

S2 Fig Simulations with an alternative parameter combination. To test the implications of alternative parameter combinations, we set the threshold protein copy number for activation to 300 copies. We found that to capture the basal activation level in experiments, *f*_*on*_ = 0.039 ± 0.003, we had to set *k*_*NFκB*_ = 9 × 10^−5^ molecules s^-1^ and *k*_*Basal*_ = 2.81 × 10^−3^ s^-1^. With these parameter combinations, we calculated *f*_*on*_ as a function of (A) *ϕ*_*PKCa*_ and (B) *ϕ*_*HDACi*_. Following the procedure in Figs 3 and 4, we recalculated the dose-response curves for (C) bryostatin-1 and (D) VPA. (E) Without adjustable parameters, our simulations (lines—mean, shaded regions—95% confidence intervals) again captured experimental observations (symbols) of the influence of using these drugs together quantitatively. Bryostatin-1 concentrations are color-coded: 1 nM (blue) or 10 nM (red).
(TIF)

S3 Fig Comparison with deterministic model. With high activation levels, obtained using *k*_*NFκB*_ = 5 × 10^−2^ molecules s^-1^ and *k*_*Basal*_ = 3 × 10^−2^ s^-1^, the time-evolution of the protein copy number predicted by our simulations (blue line) was indistinguishable from that predicted by a deterministic model (red line) of the HIV-1 latency circuit (S1 Text). The deviations (shaded region) from the mean (line) in our stochastic simulations were small and all cells were activated, yielding *f*_*on*_ = 1.
(TIF)

S4 Fig Robustness of simulations. Dependence of *f*_*on*_ on *ϕ*_*HDACi*_ for 3 different values of *ϕ*_*PKCa*_ obtained using our simulations with 2000 cells and 10 realizations for each parameter combination (squares) compared with the same simulations using 4000 cells (inverted triangles) or 20 realizations (triangles) for each parameter combination. The other parameter values are in Table 1.
(TIF)
