# Supplementary material for: Trade-off between synergy and efficacy in combinations of HIV-1 latency-reversing agents
Source: PLoS Comput Biol. 2018 Feb 16;14(2):e1006004. doi: 10.1371/journal.pcbi.1006004 (PMC5833289; doi:10.1371/journal.pcbi.1006004)
Supplement: S1 Text — (PDF) [file pcbi.1006004.s001.pdf]

Text S1. Deterministic model of the HIV-1 latency circuit

The following differential equations yield a deterministic model of the HIV-1 latency circuit in Eqs. (1)-(18) in the absence of LRAs:

$$\frac{d}{dt}\left[NF-\kappa B_c\right]=k_{NF\kappa B}-k_{ImpNF\kappa B}\left[NF-\kappa B_c\right]-\delta_{NF\kappa B}\left[NF-\kappa B_c\right] \tag{S1.1}$$

$$\begin{aligned}\frac{d}{dt}\left[NF-\kappa B_n\right]=&\,k_{ImpNF\kappa B}\left[NF-\kappa B_c\right]-k_{On}\left[NF-\kappa B_n\right][LTR]+k_{Off}[LTRNF]\\&+k_{Transact}[LTR-Tat_a]-\delta_{NF\kappa B}\left[NF-\kappa B_n\right]\end{aligned} \tag{S1.2}$$

$$\frac{d}{dt}\left[LTR\right]=-k_{On}\left[NF-\kappa B_n\right][LTR]+k_{Off}[LTRNF]+k_{Transact}[LTR-Tat_a] \tag{S1.3}$$

$$\frac{d}{dt}\left[LTRNF\right]=k_{On}\left[NF-\kappa B_n\right][LTR]-k_{Off}[LTRNF]-k_{Bind}\left[LTRNF\right][Tat_n]+k_{Unbind}\left[LTR-Tat_d\right] \tag{S1.4}$$

$$\frac{d}{dt}\left[mRNA_n\right]=k_{Basal}[LTRNF]+k_{Transact}[LTR-Tat_a]-k_{ExpmRNA}[mRNA_n]-\delta_{mRNA}[mRNA_n] \tag{S1.5}$$

$$\frac{d}{dt}\left[mRNA_c\right]=k_{ExpmRNA}[mRNA_n]-\delta_{mRNA}[mRNA_c] \tag{S1.6}$$

$$\frac{d}{dt}\left[P\right]=k_{Protein}[mRNA_c]-\delta_{Protein}[P] \tag{S1.7}$$

$$\frac{d}{dt}\left[Tat_c\right]=k_{Tat}[mRNA_c]-k_{ImpTat}[Tat_c]-\delta_{Tat}[Tat_c] \tag{S1.8}$$

$$\frac{d}{dt}\left[Tat_n\right]=k_{ImpTat}[Tat_c]-k_{Bind}\left[LTRNF\right][Tat_n]+k_{Unbind}\left[LTR-Tat_d\right]-\delta_{Tat}[Tat_n] \tag{S1.9}$$

$$\frac{d}{dt}\left[LTR-Tat_d\right]=k_{Bind}\left[LTRNF\right][Tat_n]-k_{Unbind}\left[LTR-Tat_d\right]-k_{Acetyl}\left[LTR-Tat_d\right]+k_{Deacetyl}\left[LTR-Tat_a\right] \tag{S1.10}$$

$$\frac{d}{dt}\left[LTR-Tat_a\right]=k_{Acetyl}\left[LTR-Tat_d\right]-k_{Deacetyl}\left[LTR-Tat_a\right]-k_{Transact}\left[LTR-Tat_a\right] \tag{S1.11}$$

We solved the equations using parameter values in Table 1 and with the initial conditions that all species except LTR were absent and a single copy of LTR was present.
