# Supplementary figures and images for: Trade-off between synergy and efficacy in combinations of HIV-1 latency-reversing agents
Source: PLoS Comput Biol. 2018 Feb 16;14(2):e1006004. doi: 10.1371/journal.pcbi.1006004 (PMC5833289; doi:10.1371/journal.pcbi.1006004)

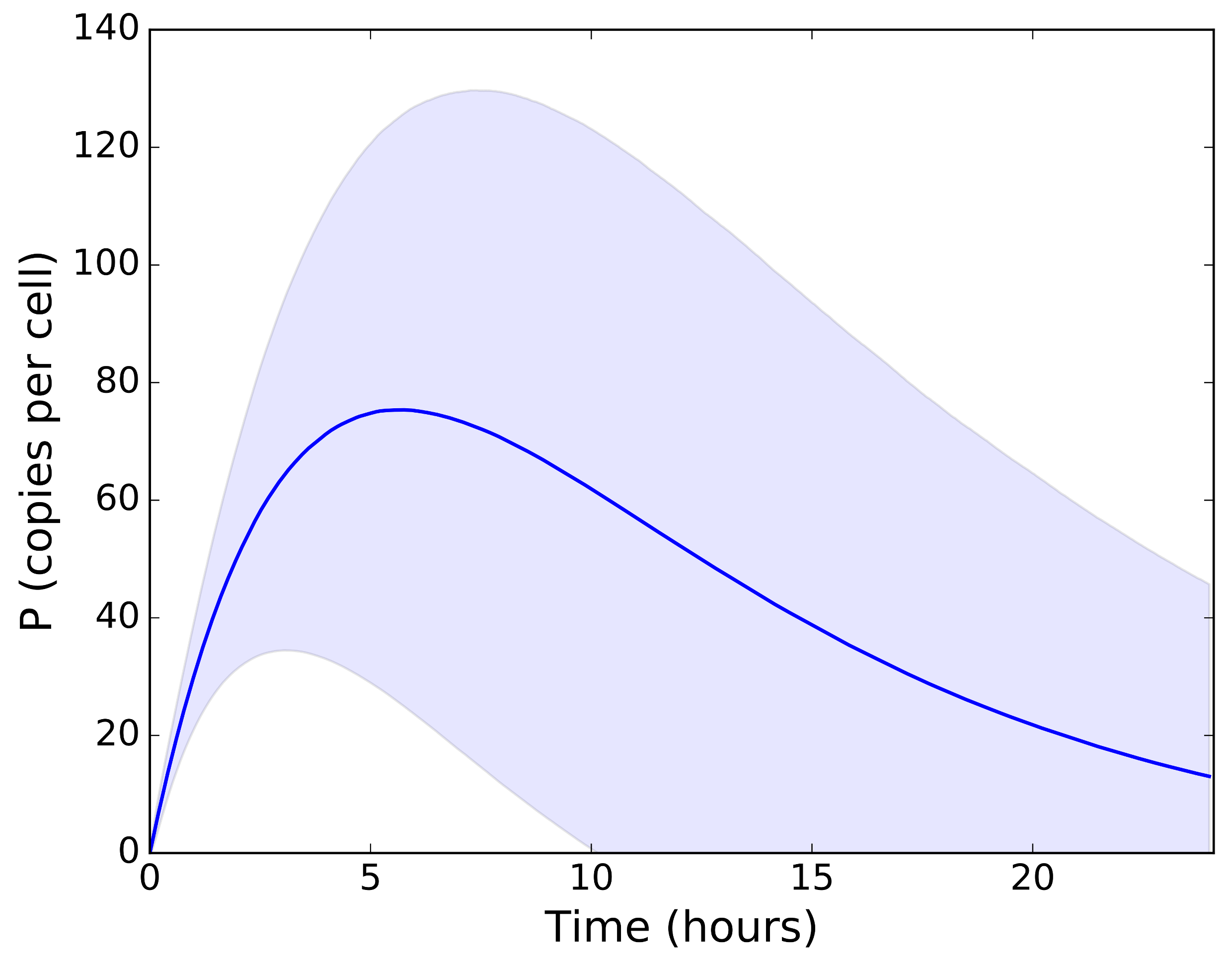

Supplement: S1 Fig — Protein copy numbers in cells lacking Tat and NF-κB obtained by simulating the reduced latency circuit, mRNAc→kProteinP+mRNAc, mRNAc→δmRNAΦ and P→δProteinΦ, which captures the protein production following a single stochastic transcription event yielding a copy of HIV-1 mRNA. The simulations were thus performed with the initial conditions mRNA(0) = 1 and P(0) = 0. The mean (line) and standard deviation (shaded region) of the resulting time-evolution of P from 104 realizations (or cells) is shown, establishing a lower bound on the threshold P for reactivation of latently infected cells. (TIF) [file pcbi.1006004.s002.tif]

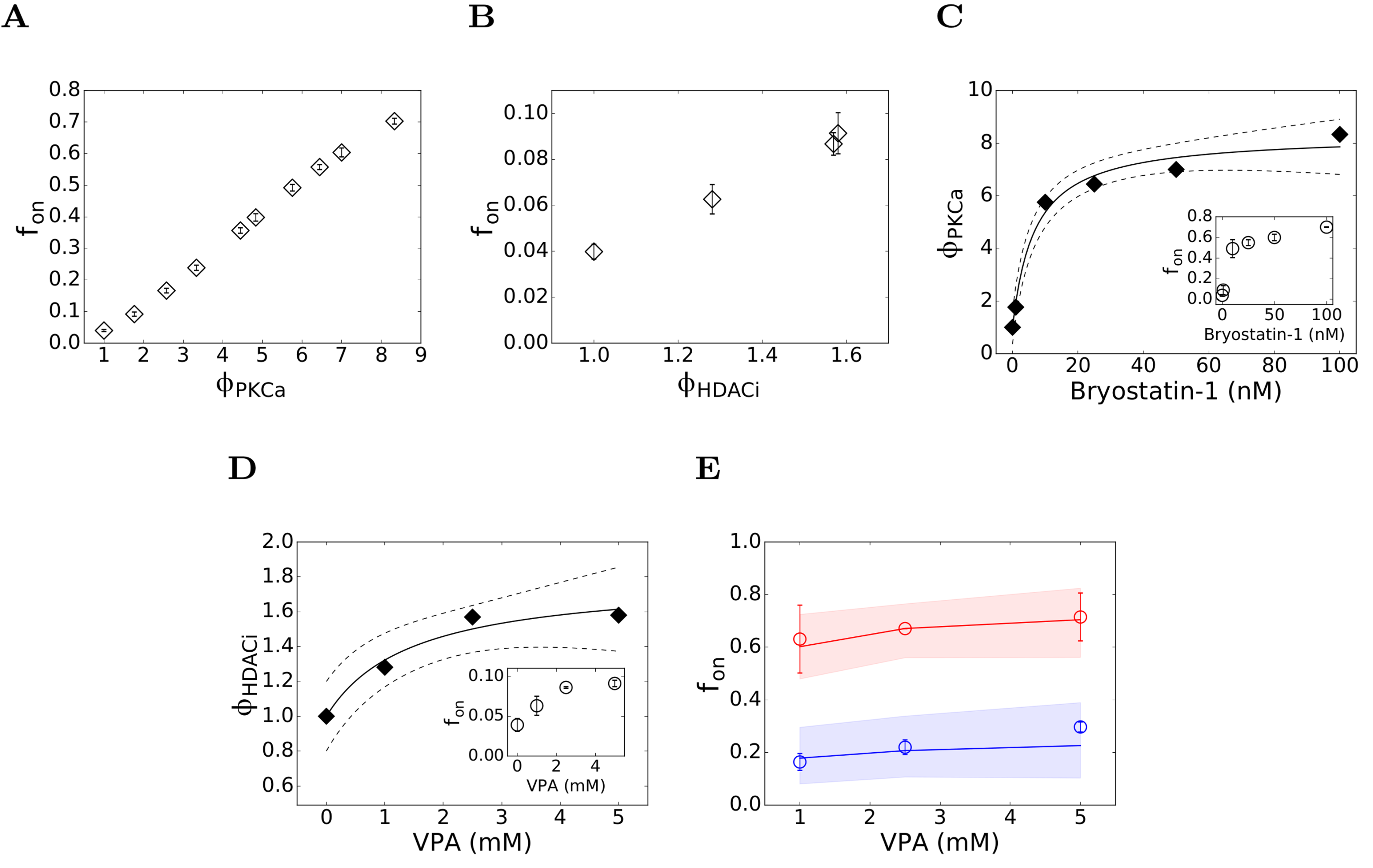

Supplement: S2 Fig — To test the implications of alternative parameter combinations, we set the threshold protein copy number for activation to 300 copies. We found that to capture the basal activation level in experiments, fon = 0.039 ± 0.003, we had to set kNFκB = 9 × 10^−5^ molecules s-1 and kBasal = 2.81 × 10−3 s-1. With these parameter combinations, we calculated fon as a function of (A) ϕPKCa and (B) ϕHDACi. Following the procedure in Figs 3 and 4, we recalculated the dose-response curves for (C) bryostatin-1 and (D) VPA. (E) Without adjustable parameters, our simulations (lines—mean, shaded regions—95% confidence intervals) again captured experimental observations (symbols) of the influence of using these drugs together quantitatively. Bryostatin-1 concentrations are color-coded: 1 nM (blue) or 10 nM (red). (TIF) [file pcbi.1006004.s003.tif]

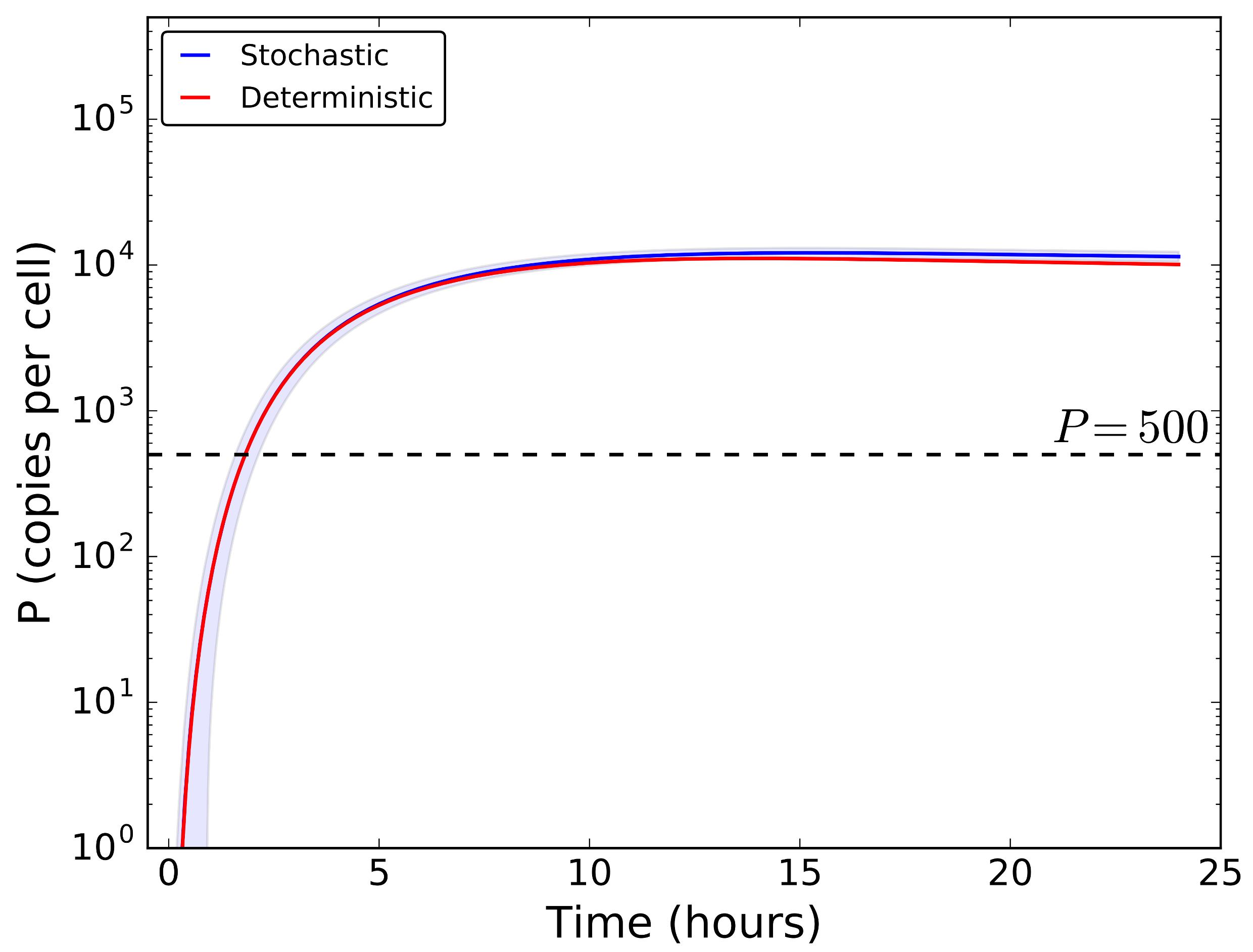

Supplement: S3 Fig — With high activation levels, obtained using kNFκB = 5 × 10^−2^ molecules s-1 and kBasal = 3 × 10−2 s-1, the time-evolution of the protein copy number predicted by our simulations (blue line) was indistinguishable from that predicted by a deterministic model (red line) of the HIV-1 latency circuit (S1 Text). The deviations (shaded region) from the mean (line) in our stochastic simulations were small and all cells were activated, yielding fon = 1. (TIF) [file pcbi.1006004.s004.tif]

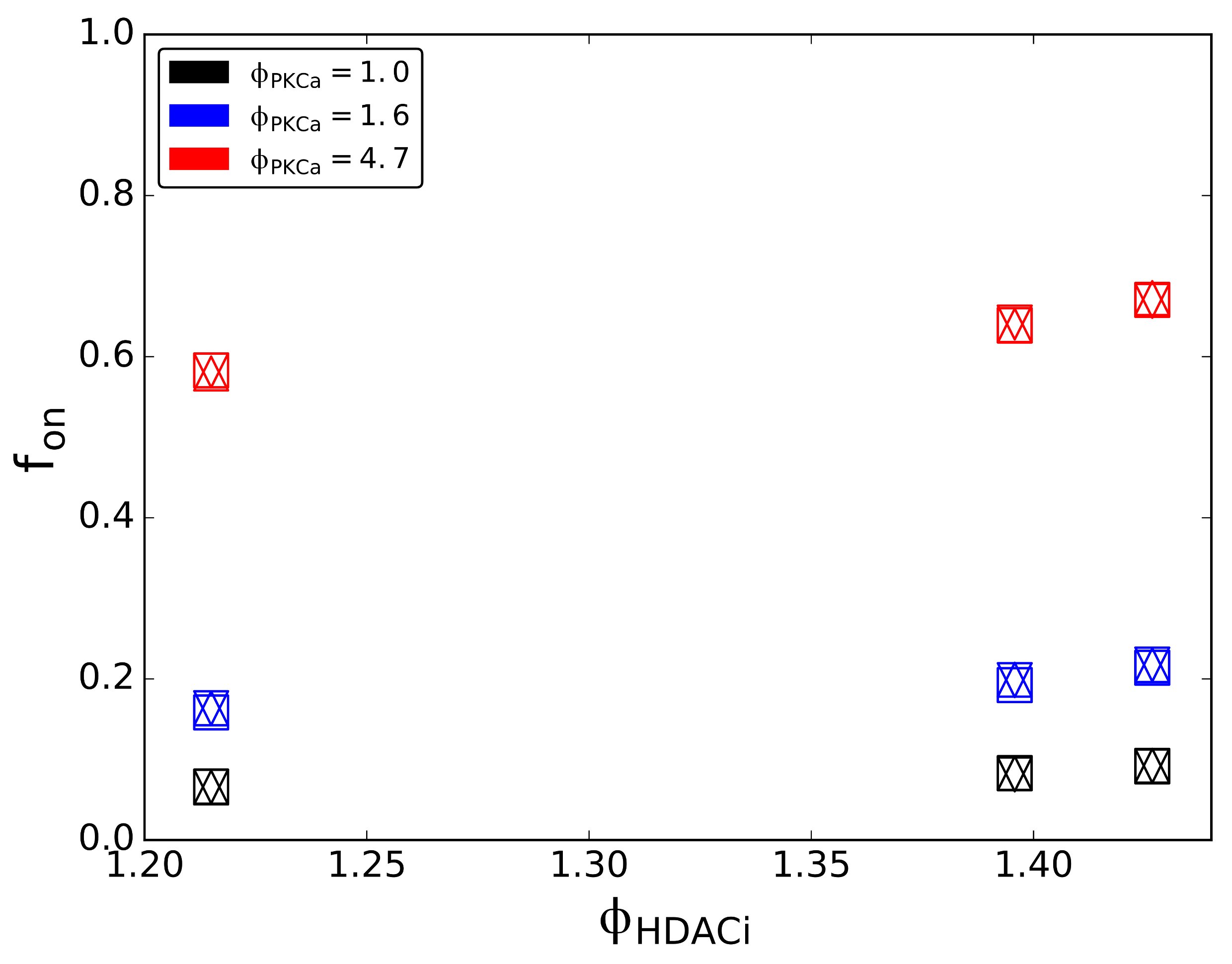

Supplement: S4 Fig — Dependence of fon on ϕHDACi for 3 different values of ϕPKCa obtained using our simulations with 2000 cells and 10 realizations for each parameter combination (squares) compared with the same simulations using 4000 cells (inverted triangles) or 20 realizations (triangles) for each parameter combination. The other parameter values are in Table 1. (TIF) [file pcbi.1006004.s005.tif]
